# Effect of a single meloxicam administration on newborn Holstein–Friesian dystocia calves: Field results from the first 10 days of life

**DOI:** 10.14202/vetworld.2022.981-985

**Published:** 2022-04-19

**Authors:** Fabian Kunz, Sait Sendag, Mehmet Yildiz, Klaus Failing, Axel Wehrend

**Affiliations:** 1 Clinic for Obstetrics, Gynecology and Andrology of Large and Small Animals with Ambulatory Service, Justus-Liebig-University, Giessen, Germany; 2 Clinic for Veterinary Obstetrics and Gynecology, University of Van YYÜ, Van, Turkey; 3 Biomathematics and Data Processing Working Group, Justus-Liebig University, Giessen, Germany

**Keywords:** calf, dystocia, nonsteroidal anti-inflammatory drugs

## Abstract

**Background and Aim:**

Calves have increased morbidity and mortality rates after dystocia. One cause is pain during birth, which reduces their colostrum intake. The administration of nonsteroidal anti-inflammatory drugs (NSAIDs) may break this causality. This study aimed to determine the consequences of a single administration of the NSAID meloxicam to dystocia calves after birth.

**Materials and Methods:**

Fifty Holstein–Friesian calves born with dystocia from four dairy cattle farms were included in this study. The animals were randomized into two groups. The animals in Group I (n=25, treatment group) received subcutaneous 0.5 mg meloxicam/kg body weight 2-8 h after birth. The animals in Group II (n=25, control group) received a control substance (Amynin^®^, bovine infusion solution, Merial) with the same volume. The newborn calves were clinically examined on the 1^st^ and 10^th^ days of life. The information regarding the days in between was gathered by questioning the farmer.

**Results:**

There was a significant difference (p=0.04) only in calves with thin, mushy fecal consistency on the 10^th^ day in the treatment group compared with the control group. Moreover, meloxicam had no effect on dystocia calves.

**Conclusion:**

Since NSAID administration did not produce a significant clinical effect, its necessity is questionable. Further studies should examine how modifying its application time would have an effect. The primary indicators of well-being, such as pain indicators in the blood, were not measured; however, these should be considered in subsequent studies.

## Introduction

The phase after birth is a high-risk time for newborn calves. Especially after dystocia (a difficult birth), calves show an increased incidence of diseases, such as diarrhea and bronchopneumonia. One of the causes is pain during birth, which reduces their colostrum intake and absorption and increases their susceptibility to infection [1].

Little attention has been given to the pain experienced by calves during dystocia, and the relationship between the pain during parturition and diseases later in life is unclear. In this context, pain/stress management and the use of nonsteroidal anti-inflammatory drugs (NSAIDs) are becoming increasingly important [2-4]. Studies on the use of NSAIDs have focused primarily on cows after dystocia [5,6]. There are only a few studies on the use of NSAIDs on calves after dystocia.

Thus, this study aimed to investigate the clinical effects of a single dose of meloxicam on newborn calves after dystocia under field conditions.

## Materials and Methods

### Ethical approval

Examinations and treatments were performed according to the standard therapeutic measures without any unnecessary harm to the animals. Approval from the Institutional Animal Ethics Committee was not required; the study did not affect the animals in excess of therapy.

### Study period and location

The study was conducted from August 2012 to August 2014. This study was carried out at four farms in the Hessen region (Germany).

### Study design

The study was conducted on 50 Holstein–Friesian calves according to the veterinary care and ethics of experimental animals. These animals originated from four dairy farms. The inclusion criteria were as follows:

- Calves born with maternal dystocia
- Expiration of the physiological gestation period

Based on clinical examination results on the day of birth, the exclusion criteria were as follows:

- Abnormalities
- Rectal temperature ≥39.5°C
- Lying down
- No milk intake.

### Randomization and medication procedures

The dystocia calves (n=50) were randomly divided into two equal groups (treatment and control). The treatment group (n=25) received meloxicam subcutaneously (0.5 mg meloxicam/kg body weight, Metacam^©^ 20 mg/mL injection solution, Boehringer Ingelheim, Germany). The control group (n=25) received an equivalent volume of Amynin^©^ (infusion solution for cattle, Boehringer Ingelheim) subcutaneously. The injection was given within the first 8 h of birth.

### Clinical examination of the dystocia calves

The newborn calves were clinically examined on the 1^st^ and 10^th^ days of life in which the following parameters were recorded:

- Posture
- Behavior
- Rectal temperature
- Abdominal rigidity
- Respiratory rate
- Color of the nasal mucosa
- Inspection of the episcleral vessels
- Auscultation of the lung and heart
- Sucking reflex
- Simultaneous auscultation and percussion of the abdomen
- Fecal quality
- Condition of the navel
- Condition of the joints

In addition, the rectal temperature was recorded on the first 5 days of life between 8:00 and 10:00 AM by a farmer. The feed or milk intake was also recorded on the first 10 days after birth. A veterinarian excluded calves with lung, intestinal, and navel diseases from the study up until day 10.

### Definition of diseases

A disturbed general condition existed when at least one parameter deviated from reference values during the final clinical examination. The lungs were considered to be diseased when abnormal auscultation sounds were heard. Similarly, the umbilical cord was considered to be diseased when pain and swelling were present.

### Statistical analysis

To determine the significance of 1 time, qualitative parameters between the treatment and control groups, two-dimensional frequency tables were created and calculated using Fisher’s exact test. The following features were compared in this test:

- Frequency of general disorders
- Incidence of lung diseases
- Incidence of intestinal diseases
- Frequency of navel diseases
- Frequency of sick animals

In addition, for 2×3 contingency tables, the Fisher–Freeman–Halton test was used. The Wilcoxon–Mann–Whitney test was used for ordinal parameters. Unique quantitative characteristics were calculated using one sample t-test. For group-time interactions, two-factor analysis of variance with repeated measurements was used. The exact Wilcoxon–Mann–Whitney test was used for qualitative progressive data with rare events. A comparison of the relative frequency of events between the treatment and control groups was also made using the exact Wilcoxon–Mann–Whitney test. The statistical programs BMDP/Dynamic (Statistical Software Manual, Volume 1 and 2. University of California Press, Berkeley, Los Angeles, Release 8.1), StatXact^®^, Version 9 (Cytel Software Corporation, Cambridge, MA, USA), BiAS for windows, and biometric analysis of samples (Version 9.08, Epsilon-Verlag, Hochheim, Darmstadt, Germany) were used for the evaluations.

## Results

### Changes in clinical parameters between the treatment and control groups on days 1 and 10 postpartum

At the time of the initial examination, the mean respiratory rate values were higher than those in the final examination. On both examination times, the calves were distributed evenly over both groups. The heart rate was also homogeneously distributed over both groups at both times. A slightly higher number of calves in the treatment group had mild-to-moderate lung sounds, but the difference was not statistically significant (Table-1). On the initial examination, enlarged navels were found in five calves, and on day 10 postpartum, enlarged navels were found in seven calves. Swollen and painful navels were diagnosed in two (postpartum day 1) and four (postpartum day 10) calves. Nevertheless, there was no significant difference in the navel findings between the groups (Table-1). The internal rectal temperatures in the treatment and control groups are presented as arithmetic mean values with standard deviation. The values measured over the investigation period showed an almost identical course at the same level. The number of calves with fever in the treatment group and the average duration of febrile periods were compared with those in the control group. In three calves in the treatment group (14%), an internal body temperature of over 39.5°C was measured, which lasted for approximately 1.7 days. Although no fever was found in the control group, there was no significant difference between the two groups. The number of calves that did not drink milk was higher, and its duration during the study period was longer in the control group. There was a reduced milk intake at the same time in both groups. Approximately 47% of the calves in the treatment group showed a thin, mushy fecal consistency on the 10^th^ day of life. This differed significantly from that of 17% of the control animals (p=0.04) (Table 2).

**Table 1:** Comparison of the frequency of lung and navel diseases between the treatment and the control groups on days 1 and 10 postpartum (There were no statistically significant differences in the lung disease and navel findings between the groups).

Groups	With lung diseases (%)	Without lung diseases (%)	With navel disorders (%)	Without navel disorders (%)
Treatment	7 (33)	14 (77)	7 (33)	14 (77)
Control	6 (21)	23 (79)	7 (24)	22 (76)
Total	13 (26)	37 (74)	14 (28)	36 (72)

**Table 2:** Comparison of fecal consistency between the treatment and control groups on days 1 and 10 postpartum.

Groups	Animals with fluid fecal consistency		Animals with thin mushy fecal consistency		Animals with medium mushy fecal consistency		Animals with solid fecal consistency	
			
	Day 1 (%)	Day 10 (%)	Day 1 (%)	Day 10 (%)	Day 1 (%)	Day 10 (%)	Day 1 (%)	Day 10 (%)
Treatment	1 (5)	2 (10)	2 (10)	10 (47)^a^	18 (85)	9 (43)	0 (0)	0 (0)
Control	2 (7)	2 (7)	6 (21)	5 (17)^b^	19 (65)	21 (72)	2 (7)	1 (4)
Total	3 (6)	4 (8)	8 (16)	15 (30)	37 (74)	30 (60)	2 (4)	1 (2)

^a-b^ Fecal consistency differed significantly (p=0.04) between the groups on the 10^th^ day of life.

### Incidence of diseases between the treatment and control groups on days 1 and 10 postpartum

The general condition of the animals from both groups was equally disturbed. In total, 46% of the 50 examined calves showed a disturbed general condition. Lung diseases occurred in 33% of the treated calves and in 21% of the control animals. Navel disorders occurred in 33% of the treated and in 24% of the control animals (Table-1). Approximately 34% of the calves remained on the farm, whereas 12% died before the 60^th^ day of life. Of these, 5% and 18% originated from the treatment and control groups, respectively.

## Discussion

Drug treatment of farm animals is the world’s biggest animal welfare issue, and this sector is getting stronger due to new developments in the pharmaceutical industry. Conversely, rising production costs also promote the need for new methods and medications. Accordingly, researchers and veterinary practitioners have to deal with modern prophylaxis strategies and new therapy methods.

In this context, pain management and the use of NSAIDs are becoming increasingly important [2-8]. Birth pains are one of the most intense pains [9,10]. Difficult births cause postpartum illnesses with considerable pain stimuli, leading to performance losses in cows and calves [11,12]. Only a few studies focused on the control of pain and inflammation in newborn calves after dystocia. This investigation was based on a hypothesis that the administration of the NSAID meloxicam in dystocia calves leads to a reduction in pathological clinical parameters, making a positive effect on performance. According to a survey among veterinarians in the United Kingdom and North Ireland by Huxley and Whay [13], 39% of practicing veterinarians use NSAIDs on calves after dystocia. In addition, according to a survey by Laven *et al*. [14], 14% of calves are treated with NSAIDs after dystocia. Nevertheless, no objective statements can be made in practice regarding the effects of NSAIDs on calves after birth. There are only five scientific studies [15-19] that mentioned the clinical effects of NSAIDs on newborn calves, stating that its use led to positive effects on performance and health parameters [8]. Todd *et al*. [19] demonstrated a positive effect of meloxicam in a double-blind study with 56 male Holstein–Friesian calves suffering from neonatal diarrhea. Half of the calves with diarrhea received 0.5 mg/kg body weight of meloxicam subcutaneously on the 1^st^ day, whereas the other half received an ineffective placebo. Their body weight, intake of milk, starter ration and water, and duration of the disease were recorded. Compared with the control group, the treatment group ingested 5.3 times more milk from the 10^th^ day of life, drank more water, showed higher daily weight gains due to the 3.19 times earlier intake of the starter ration, and had a disease course shortened by 65%. Thus, the calves treated with meloxicam reached the weaning weight earlier. Hence, it was concluded that the treatment of neonatal diarrhea with meloxicam is an effective supportive therapy [19]. Another study by Murray [15] evaluated the risk factors and management programs for newborn calves in five independent trials. In a partial study with 284 calves, the effects of meloxicam on vitality, sucking reflex, milk intake, daily growth, and calf health were evaluated. In another study with 842 calves, Murray [16] recorded the effects of meloxicam on vitality, passive immunoglobulin transfer, daily growth, and calf health of dystocia calves. In both studies, half of the calves were randomized to receive 0.5 mg/kg body weight meloxicam subcutaneously immediately after birth, whereas the other half received an ineffective control substance. Overall, Murray [15] demonstrated that the dystocia calves had increased acidosis (blood lactate values higher by 2.93 mmol/L), less motivation to get up, weakened sucking reflexes, and reduced vitality. A single treatment with meloxicam resulted in increased vitality and improved sucking reflex in both studies [15,16]. Long-term effects, such as increased intake of daily milk quantities, faster weight growth, and lower disease rates, were also observed after meloxicam treatment in both studies [15,16]. No effects from meloxicam could be registered on the passive immunoglobulin transfer in the calf’s intestine. Moreover, according to the visual appearance, initiation of movement, general responsiveness, oxygenation, heart and respiration rates data on calves, Murray *et al*. [17] reported that meloxicam treatment after calving had a positive effect on their overall health. In the present study, meloxicam had no positive effect on dystocia calves. The only significant difference between the treatment and control groups was the difference in fecal consistency on the 10^th^ day of life postpartum. This result is surprising and should be interpreted cautiously since all further results showed no significant differences. In addition, this result contradicts the studies of Todd *et al*. [19] and Murray [15]. Todd *et al*. [19] chose the same dosage without making this observation. Further studies on the effects of meloxicam on calves after dystocia are recommended. It is also suspected that NSAIDs cause circulatory disorders in calves, especially in the gastrointestinal tract and kidneys, by inhibiting prostaglandin synthesis. Klahr *et al*. [20] and Agostiniani *et al*. [21] demonstrated kidney function impairments by administering NSAIDs to human neonates. No studies on calves are available in this regard. Future studies that also consider blood biochemical values will contribute significantly to the evaluation of the study results.

## Conclusion

Meloxicam had no positive effect on dystocia calves during the first 10 days of life. The collected results were clinical and performance parameters in newborn dystocia calves. The primary indicators of well-being, such as pain indicators in the blood, were not measured, but these should be considered in subsequent studies.

## Authors’ Contributions

FK, SS, and MY: Performed the experimental work, interpreted the data, and drafted the manuscript. KF: Data interpretation. AW: Project advisor and reviewed the manuscript. All authors read and approved the final manuscript.
